# Keyword Index for Volume 93

**DOI:** 10.1038/sj.bjc.6602898

**Published:** 2005-12-06

5-aminolevulinic acid 1137

5-fluorouracil 993

5-fluorouracil modulation 896

5-FU 185

5T4 670

ablation 890

abnormal p53 protein expression 946

actigraphy 1202

acute lymphoblastic leukaemia 379

adenocarcinoma 450, 1301

adiponectin 811

adjuvant chemotherapy 627, 884, 896

adjuvant endocrine therapy 1319

adolescence 817

adriamycin 544

advanced breast cancer 515

advanced head and neck cancer 884

advanced pancreatic cancer 185

adverse skeletal events 633

aetiology 842

affinity 1257

Africa 1068

age adjustment 372

age–period–cohort 1077

AKT 1372

all-cause mortality 392

alternative medicine 538

amplification 699

anaemia 224

angiocidin 662

angiogenesis 613, 781, 793, 855, 1128, 1350, 1356

angiotensin I/II 425

angiotensin-converting enzyme 602

anoikis 233

anthropometry 1310

antiangiogenic therapy 876

antibody 1085

antibody response 458

anti-Fas monoclonal antibody 544

anti-late-diarrhoeal program 1341

antisense RNA 216

apoptosis 1, 70, 208, 233, 973, 1157, 1356

array CGH 699, 1191

arterial infusion chemotherapy 557

association 493

ATM heterozygosity 260

aurora 719

basal cell carcinoma 597

Bayesian methods 1077

betel quid 602

biobank 834

biomarkers 735

BK virus 1305

bladder 346

bladder cancer 804, 1182

bleomycin 1209

blood flow 98

body fatness 817

body mass 1057

body mass index 807, 811, 1310

bone metastases 633

bone morphogenetic proteins 850

brain tumours 152, 781

*BRCA1* 287

*BRCA2* 287

breast cancer 167, 287, 293, 364, 392, 406, 520, 590, 627, 694, 699, 793, 817, 924, 973, 1005, 1062, 1128, 1152, 1168, 1215, 1295, 1372

breast cancer mortality 392

breast carcinoma 1046

breast neoplasm 582, 1122

breast tumour xenografts 1152

breast-conserving surgery 520, 1122

breastfeeding 379

buccal biopsy 208

burnout 1092

CA19-9 195, 740

cachexia 774

calcium 639, 1364

calcium sensing receptor 1364

calprotectin 639

cancer 478

cancer cachexia 425

cancer of the small intestine 807

cancer of the testis 909

cancer predisposition 260

cancer registries 159, 372

cancer vaccine 1250

capecitabine 510, 1117

carbonic anhydrase IX 1267

carboplatin 1209

carcinogenesis 331

carcinoid 1144

carcinomas 346, 464

cardia 859

case–control 167

case–control study 1057

CD146 793

CD34+ cell 855

CD4 and CD8 lymphocytes 435

cell cycle 939

cervical cancer 376, 862, 1077

cervix 1301

chemoprevention 735

chemoradiotherapy 652, 1117

chemotherapy 29, 54, 224, 233, 279, 302, 483, 740, 744, 909, 977

chemotherapy resistance 510

chemotherapy response 515

child 379

childhood 817

childhood acute lymphoblastic leukaemia 1307

childhood acute myeloid leukaemia 1388

childhood and adolescent cancer 1038

childhood chronic diseases 1053

children 412

chimeric immune receptor 1085

chromosomal radiosensitivity 1038

CIN 248

circadian function 1202

circulating endothelial cells 793

cisplatin 178, 529, 678, 763, 909, 1112, 1117, 1209, 1341, 1356

ckit 107

clinical 890

clinical guidelines 1122

clinical trials 41

clonogenic assay 70

coffee 607

cohort studies 804, 807, 1053, 1310

colon cancer 338, 1356

colon carcinoma 1364

colonic cancer 1062

colorectal adenomas 954

colorectal cancer 1, 399, 510, 744, 799, 896, 1230, 1236

comorbidities 1098

comparison 552

contrast agent 131

C-reactive protein 639

CRM 529

CT antigen 458

cutaneous melanoma 597

CXCR4 924

cyclin A 515

cyclin D1, D2, D3 338

cyclooxygenase-2 954

cytarabine 1388

cytidine deaminase 35

cytochrome P450 973

cytology 1175

cytoskeleton 499

cytotoxic 979

darbepoetin alfa 224

DCE-MRI 979

Decoy receptor 3 544

delay 627

deletion 699

dendritic cell 749

deoxynucleoside analogues 1388

dependence receptor 1

deprivation 622

diabetes mellitus 1310

diagnosis 399, 905

differentiation 338, 450, 1364

disease grade 248

disease-free survival 453

DNA microarray 1182

DNA replication licensing 1295

DNA sequence 1068

DNA vaccination 1250

DNA-PK 1011

docetaxel 173, 999

dose density 7

doxil 46

doxorubicin 89

*DRM/Gremlin* (*CKTSF1B1*) 1029

drug delivery 224

drug resistance 483

drug therapy 735

ductal carcinoma *in situ* 1122

dysadherin 1382

early breast cancer 1319

early-onset breast cancer 260

E-cadherin 1364, 1382

ectodomain shedding 1267

ED1 and ED2 macrophages 435

effectiveness 376

EGFR 107, 124, 915, 1334

EGFRvIII 915

elastase 1024

elderly 770

ELISPOT 248

embryonal carcinoma 1382

EMEA 504

end points 504

endometrial cancer 999, 1062

endostatin 1024

endothelial progenitor cell 855

endothelin-1 98

EORTC 387

ephrin-A1 933

EPIC 1305

epidemiology 811, 838, 842

epidermal growth factor receptor 355

epigenetic inactivation 850

epirubicin 406

EPR effect 678

erbB-2 7

erythropoietin 224, 1350

ET_A_ and ET_B_ receptors distribution 98

ET_B_ agonist IRL 1620 98

etoposide 216, 1209

etoposide phosphate 60

ETS 933

evaluation 862

expression profiling 924

extragonadal germ cell tumour 412

Fab fragment 1257

FAK 694

familial cancer 260

family history 825, 1307

farnesyltransferase inhibitor 1222

Fas 544

Fas ligand 544

FDA 504

feasibility 208

feasibility study 884

febrile neutropenia 1324

fertility 200, 859

fine-needle aspiration 1175

first-line 190

fixed dose rate infusion 35

flk-1 1005

flt-1 1005

fluorouracil 190, 1236

fluvastatin 319

folate 639

follicular neoplasms 1175

follicular thyroid carcinoma (FTC) 144

free fatty acids 639

galectin-3 1175

gastric cancer 688, 986, 1395

gastrocnemius muscle 774

gefitinib 23, 915, 1334

gemcitabine 29, 185, 195, 319, 406, 770, 1112

gemcitabine pharmacokinetic 35

geminin 1295

gene expression 1182, 1277

gene expression profiling 310

gene microarray 483

gene transfer 441

genetic epidemiology 167

genetic instability 719

genetic polymorphism 167

germ-cell tumour 909

gestational trophoblastic neoplasia 620

Gleason grade 1019

glioma 124

glutathione-S-transferase 216

goserelin 647

GOT1 1144

grade of tumour 946

growth arrest 1364

GTPase 499

guideline adherence 520

guidelines 387

haplotypes 954

hazard ratio 607

HB-94 538

head and neck cancer 1117

healing 538

hENT1 1388

hepatocellular carcinoma 458, 557, 607

HER-2 107, 1250

HER-2 overexpression 552

HER3 1334

HHV8 834

HIF 346

HIFU 890

high-dose chemotherapy 412, 620

high-dose palliative radiotherapy 652

high-risk HPV 862

histamine dihydrochloride 757

histology 364, 1046

HL60 human promyelocytic leukaemia 89

Hodgkin’s lymphoma 571

homocysteine 639

Hong Kong 1077

hormone receptor status 946

hormone receptors 364

hormone replacement therapy 582

hormones 200

*HPP1* 1029

HPV 575, 1068, 1301

HPV16 248

HT29 338

hTERT 116, 152

human 116

human breast cancer 946

human papillomavirus 116, 946

hyaluronidase 81

hypermethylation 1395

hypoxia 224, 346, 1128, 1267, 1356

ID1 933

IFNAR2 557

ifosfamide 178

*IL1-RN* 493

immortalisation 116

immune response 435

immunofluorescence 834

immunoglobulin 1257

immunohistochemistry (IHC) 107, 144, 453, 464, 478, 688, 1019

immunomodulatory 613

immunoprevention 1250

immunotherapy 458, 670, 749

*in vitro* fertilisation 1053

infection 379

inflammation 331, 493

inherited predisposition 1038

insulin 639

integrins 167

interferon alpha 441

interleukin-2 273, 757

intermediate and poor prognosis metastatic germ cell tumours 1209

internalisation 1257

interstitial fluid pressure 81

interstitial laser thermotherapy 435

investigation 905

iressa 915

irinotecan 763, 1106, 1230, 1341

isoflavones 15

Japan Collaborative Cohort Study (JACC Study) 607

K-ras 355

*k-ras* mutation 850

KCNH2 781

KCNH3 781

KDR 1005

keratin 1182

keratinocytic cancer 597

Ki-67 939, 1295

kidney 890

*K*^trans^ 979

Kv 11.1 781

Kv 12.2 781

laser capture microdissection 1301

LDH 273

lenalidomide 613

leptin 811

leukaemia 379, 973

liposomal doxorubicin 81

liquid-based cytology 242, 575

liver 890

local neoplasm recurrence 1122

local vegetarian food 1057

locally advanced rectal cancer 993

LoVo39 338

low-risk 1324

[^177^Lu-DOTA^0^-Tyr^3^]-octreotate 1144

lung adenocarcinoma 355

lung cancer 652, 719, 825, 905, 1329

lymph node metastasis 924, 1128

lymph node micrometastasis 688

lymphangiogenesis 1168

lymphatic invasion 688

lymphatic microvessel density 1168

lymphatic tumour dissemination 1168

lymphatic vessel invasion 1168

lymphoma 1062, 1382

LYVE-1 1168

magnetic resonance imaging 131

major depression 1329

malignant gliomas 152

malignant lymphoma 418

malignant transformation 1285

mammary tumour 1137

mammography 590

mantle cell lymphoma (MCL) 939

marker tumour 387

marketing approval 504

mastectomy 287, 1122

matched case–control study 418

matched pairs 694

maximum likelihood estimation 1077

MCF-7 538, 1005, 1152

MCM 1295

Mcm2 1295

melanoma 216, 933

meningiomas 152

menopausal status 15, 582

meta-analysis 825

metabolism 1011

metachronous colorectal cancer 472

metalloproteases 1267

metaregression 1215

metastases 552

metastasis 1244, 1277

metastatic gastric cancer 190

metastatic germ cell cancer 178

metastatic melanoma 273

metastatic stage 450

methylation 850, 1029

MGMT 529, 1152

microarray 346

microenvironment 302

microsatellite instability 472

microvascular pressure 81

minichromosome maintenance protein 6 (MCM6) 939

minimal residual disease 565

minor depression 1329

mitochondrial DNA 331

mitogen-activated protein kinase 1364

mitomycin C 510

molecular diagnostics 565

monoclonal antibody 131

monocytes 273, 855

mTOR 1372

mts1 protein 1277

mucin 1257

multicellular resistance 302

multidisciplinary teams 977, 1092

multidrug resistance 46, 89

multidrug resistance protein 216

multiple myeloma 70, 613, 834

muscle wasting 425

mutation 915, 1244

myelodysplastic syndromes 613

*n*-3 fatty acids 639

NADPH cytochrome *P*450 reductase 89

natural killer cell 441

NCI 387

needle biopsy 478

neoplasia 499, 735

neoplasm 859

nested case–control study 834

neuroblastoma 310

neuroma, acoustic 842

neuronal markers 1197

neurotoxicity 678

neutrophils 273

never-smoker 23

NF-*κ*B 1019, 1285

non-Hodgkin 1062

non-Hodgkin’s lymphoma 159, 811

nonmelanoma skin cancer 597

non-small-cell lung cancer 23, 29, 450, 453, 850, 977, 1098, 1106, 1202, 1334, 1341

non-small-cell lung carcinoma 137

Norway 807

Notch 709

NSCLC 763, 770, 1157

NU7026 1011

nuclear factor κB 70

nude mice 1005

nurse-managed care 41

nutrition 735

obesity 582, 1062, 1310

oesophageal cancer 107, 1112

oestrogen 392, 859

oestrogen plus progestin 392

oestrogen receptor 1046

omega-3 fatty acid 1329

oncofoetal antigen 670

oncofoetal fibronectin mRNA 565

oncology drugs 504

oral antibiotics 1324

oral precancerous lesions 602

oral squamous cell carcinoma 1250

organised programme 376

osteopontin (OPN) 453

ovarian adenoma 709

ovarian cancer 60, 973

ovarian carcinoma 709

ovarian neoplasms 647

ovarian surface epithelium 116

overall survival 453

oxaliplatin 29, 190, 993, 1230

oxidative stress 331, 757

p16 933

p53 242, 355

p53 mutations and ERB-2 expression 946

p53 pathway 124

p70S6K 1372

paclitaxel 178, 1106

paediatric phase I/II 529

palliative 54

palliative benefit 652

pancreas 1024

pancreatic cancer 35, 195, 441, 740, 1062

pancreatic cancer cells 319

pancreatic neoplasms 1310

papillary thyroid carcinoma (PTC) 144

participation in sports 817

pathological complete response 406

patient mortality 946

patient preferences 1319

PDK-1 1372

pegylated liposomal doxorubicin 46

pendrin 144

peritoneal metastasis 986

P-glycoprotein 973

pharmacodynamics 876

pharmacokinetics 876, 1011, 1222

pharmacology 173

phase I clinical trial 876

phase I study 1222

phase II 999, 1112

phosphorylation 1372

photodynamic detection 1137

plasminogen activator inhibitor-1 799

polymeric micelle 678

polymorphisms 954

polyubiquitin 662

portal vein thrombosis 557

predictive factors 7

premenopausal 817

prenatal origin 1307

preoperative chemoradiation 993

prevention 287

primary breast cancer 552

primary chemotherapy 406

primary health care 399

probability 1244

prognosis 7, 124, 137, 152, 450, 515, 627, 740, 838, 933, 1098

prognosis marker 924

prognostic 387

prognostic factors 273

prognostic model 273

progression 242

proliferation 208, 338, 515, 939

prospective studies 986, 1310

prostate 478

prostate cancer 493, 633, 749, 1019, 1285, 1305

prostate neoplasm 1057

proteasome expression 425

protein beam 849

protein degradation 774

proteosome 662

protoporphyrin IX 1137

PSA 1285

PSC 833 46

psychological distress 622

psychological well being 1092

*PTEN* 124

public health 862

quality of life 200, 1202, 1236

quantitative RT–PCR 144, 799

quiescent 302

Rad51 137

radiation 590, 699

radiotherapy 849, 977

raltitrexed 1230

randomised 279, 757, 862

randomised controlled trial 41

ras effectors 319

*RASSF2* 1395

rat carcinosarcoma 98

rational 54

Rb1 pathway 124

reactive metabolite(s) 89

real-time PCR 152, 793, 1301

real-time quantitative RT–PCR 986

real-time RT–PCR 565

recurrence of rectal carcinoma 131

redox cycling 89

referral and consultation 399

regional variation 520

regions of imbalance 1191

regulatory 504

relative risk of death 571

REMARK 387

renal cell carcinoma 670, 757

reproductive factors 364

rest/activity function 1202

retinoid 310

retinoid signalling 310

reverse transcription–polymerase chain reaction 688

risk 838

risk assessment 590

RT–PCR 1157

*RUNX3* 1029

rural 1068

S-1 884

S100A4 1277

salvage chemotherapy 178

salvage therapy 412

scaffold 499

screening 376, 862, 1077

season of diagnosis 571

second primary cancers 159, 418

second-line treatment 763

SEER 1046

seminoma 1382

sentinel node biopsy 520

septin 499

sequential 54

serum phospholipids 1329

sex hormones 859

side effects 744

signal transduction 70

signalling 915

SIL 1068

single-strand conformation polymorphism 355

skin neoplasms 597

sleep quality 1202

small-cell lung cancer 1197

smoking 355, 1310

smoking cessation 1310

SNPs 493, 811

social difficulties 622

somatostatin receptors 1144

soybean products 15

specific induction 694

spheroid 302

squamous cell carcinoma 597

squamous cell head and neck cancer 279

stage 1046

stem cells 719, 855

SUIT-2 1024

superparamagnetic iron oxide 131

surgery 287

surrogate end point 195

surrogate markers 1215

survival 124, 372, 977, 1215

symptoms 905

synchronous colorectal cancer 472

synergism 319

systematic review 293, 825

T cells 248

TACE/ADAM17 1267

Taiwan 1057

tamoxifen 647

taxanes 173, 293

taxol 406

taxotere 173

T*β*RII 1157

telephone 842

temozolomide 529, 1152

testicular cancer 200, 1307

testicular tumours 1382

testis-specific protein Y-encoded 458

TGF*β* 1029

TGF-*β*1 1157

thalidomide 613

therapy 1144

thrombosis 909

thyroglobulin mRNA 565

thyroid 464, 1175

thyroid cancer 565

thyroid neoplasm 1277

thyroid transcription factor 1 450

timing 627

tissue distribution 81

tissue microarray 137, 478, 1019

TNF*α* 1024

toenail selenium 804

tolerability 208

topoisomerases 54, 60

topotecan 60

toxicity 1230

transcription factor 1285

transgenic mice 1137

treatment 552

treatment efficacy 744

triacylglycerol 639

trial conclusion 41

Tsp-1 933

tumorigenicity 1157

tumour 302, 979

tumour growth 1350

tumour marker 195

tumour oxygenation 224

tyrosine kinase inhibitor 1334

UbcH10 464

ubiquitin-proteasome proteolysis 774

UFT 770

UK practice 1324

ulcerative colitis 331

unselected 1068

uracil–tegafur 770

urothelial cancer 544

urothelial carcinoma 242

uveal melanoma 1191

vaccination 1068

valspodar 46

variability 208

vascular disruptive agents 979

vascular endothelial growth factor receptor 233

vascular resistance 98

VEGF 107, 924

venous thromboembolism 838

vincristine 1209

viral load sensitivity 575

vitamin D 571

voltage-gated ion channels 1197

waiting times 905

weight gain 582

weight history 582

Western blot 144

